# Supplementary material for: Reduced Type 2 Innate Lymphocyte Cell Frequencies in Patent Wuchereria bancrofti-Infected Individuals
Source: Pathogens. 2023 Apr 30;12(5):665. doi: 10.3390/pathogens12050665 (PMC10223000; doi:10.3390/pathogens12050665)
Supplement: Supplementary file 1 [file pathogens-12-00665-s001.zip › pathogens-2234592-supplementary.pdf]

Supplementary Material

Table S1. Lineage antibodies.

Fluorochrome	Antigen	Clone
FITC	CD3	OKT3
FITC	CD4	RPA-T4
FITC	CD1a	HI149
FITC	CD14	61D3
FITC	CD16	3G8
FITC	CD19	HIB19
FITC	CD34	581
FITC	CD94	DX22
FITC	CD303a	201A
FITC	FceR1 alpha	AER-37 (CRA1)

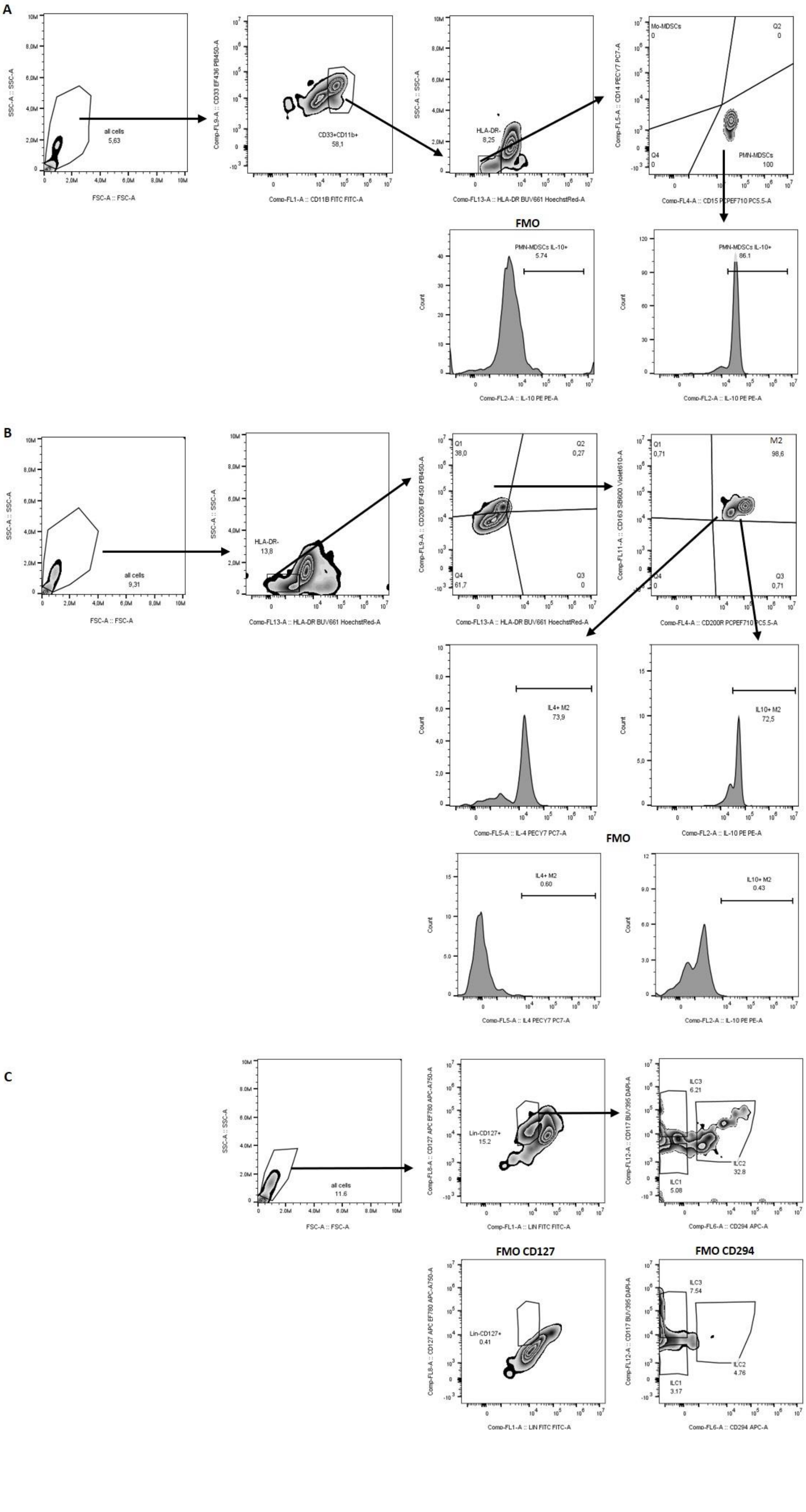
A
all cells
5,63
FSC-A :: FSC-A
SSC-A :: SSC-A
CD33+CD11b+
58,1
Comp-FL1-A :: CD11B FITC FITC-A
Comp-FL9-A :: CD33 EF436 PB450-A
HLA-DR-
8,25
Comp-FL13-A :: HLA-DR BUV661 HoechstRed-A
Mo-MDSCs
0
Q2
0
Q4
0
PMN-MDSCs
100
Comp-FL4-A :: CD15 PCPEF710 PC5.5-A
Comp-FL5-A :: CD14 PECY7 PC7-A
FMO
PMN-MDSCs IL-10+
5.74
PMN-MDSCs IL-10+
86.1
Count
Comp-FL2-A :: IL-10 PE PE-A
B
all cells
9,31
HLA-DR-
13,8
Q1
38,0
Q2
0,27
Q4
61,7
Q3
0
Comp-FL9-A :: CD206 EF450 PB450-A
Q1
0,71
M2
98,6
Q4
0
Q3
0,71
Comp-FL4-A :: CD200R PCPEF710 PC5.5-A
Comp-FL11-A :: CD163 SB600 Violet610-A
IL4+ M2
73,9
IL10+ M2
72,5
Comp-FL5-A :: IL-4 PECY7 PC7-A
FMO
IL4+ M2
0.60
IL10+ M2
0.43
Comp-FL5-A :: IL4 PECY7 PC7-A
C
all cells
11.6
Lin-CD127+
15.2
Comp-FL1-A :: LIN FITC FITC-A
Comp-FL8-A :: CD127 APC EF780 APC-A750-A
ILC3
6.21
ILC2
32.8
ILC1
5.08
Comp-FL6-A :: CD294 APC-A
Comp-FL12-A :: CD117 BUV395 DAPI-A
FMO CD127
Lin-CD127+
0.41
FMO CD294
ILC3
7.54
ILC2
4.76
ILC1
3.17

Supplementary Figure S1. Gating strategy for MDSCs, M2 macrophages and ILC populations. Peripheral blood cells were stained with fluorophore-conjugated anti-human (A) CD11b, CD33, HLA-DR, CD14, CD15, IL-6 and IL-10 monoclonal antibodies to detect MDSC subsets, (B) HLA-DR, CD206, CD163, CD200R, IL-4 and IL-10 monoclonal antibodies to detect M2 macrophages and (C) CD294, Lin cocktail [CD1a, CD3, CD4, CD14, CD16, CD19, CD34, CD94, FceR1, CD303 (BDCA-2)], CD127, CD117 and CD294 monoclonal antibodies to detect ILC subpopulations according to the presented gating strategies.

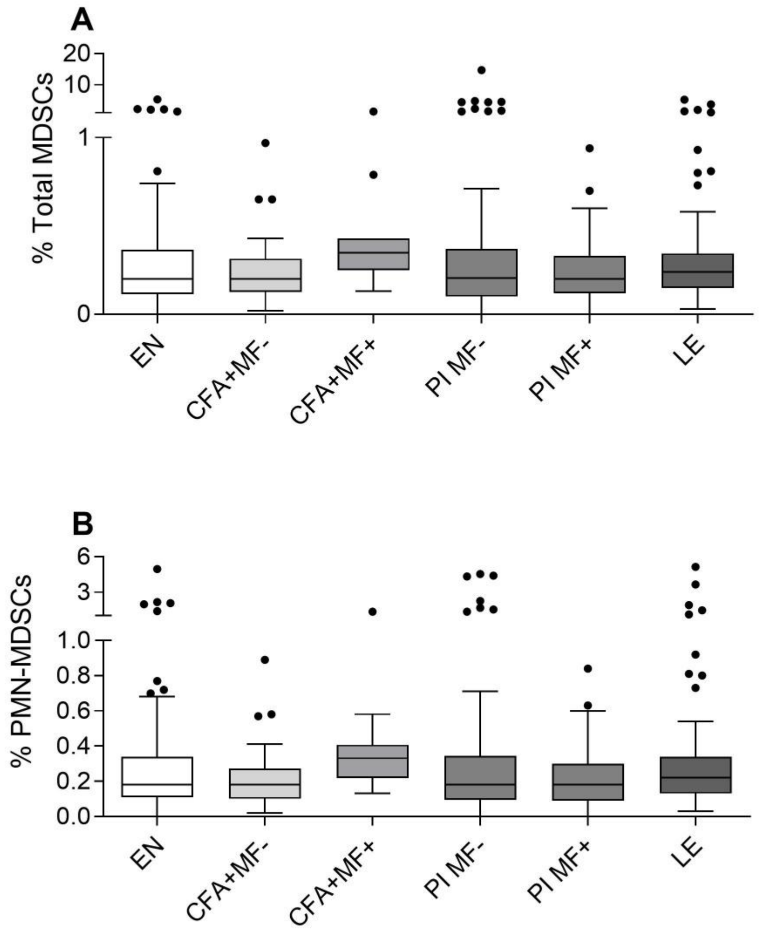

Supplementary Figure S2. MF status of PI group does not influence peripheral PMN-MDSC frequencies. To analyze if MF can influence MDSC subsets after start of treatment, the previously infected (PI) group was stratified according to presence or absence of MF after implementation and intensification of MDA in 2001 and 2009, respectively. Peripheral whole blood cells from endemic normal (EN; *n* = 89), latent (CFA+MF-; *n* = 54) and patent (CFA+MF+; *n* = 14) *Wuchereria bancrofti*-infected, as well as previously infected individuals without MF (PI MF-; *n* =86) or with MF (PI MF+; *n* = 31) before intensification of MDA programs and individuals that suffer from lymphedema (LE; *n* = 85) were analyzed for frequencies (%) of (A) total MDSCs (CD11b+CD33+HLA-DR-) and (B) PMN-MDSCs (CD11b+CD33+HLA-DR-CD14-CD15+). Graphs show box whiskers with median, interquartile ranges and outliers (dots). Statistical significances between the indicated groups were obtained after a Kruskal-Wallis-test followed by a Dunn‘s multiple comparison post hoc analysis.

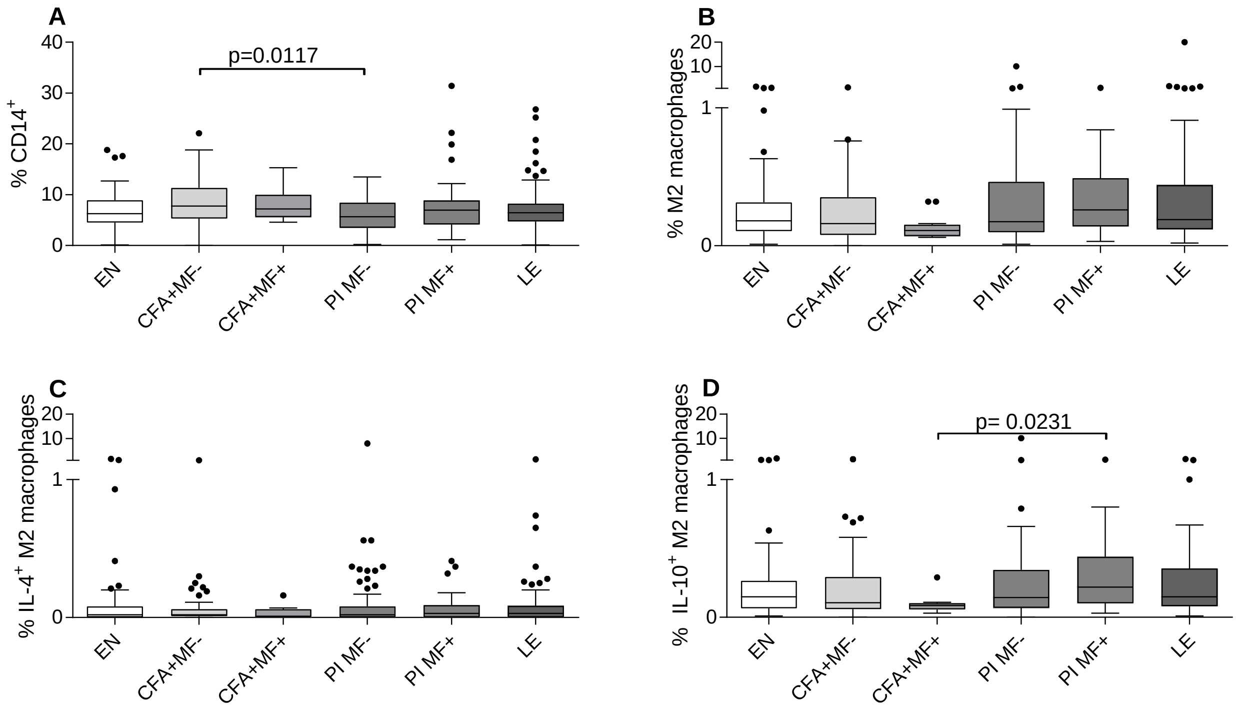

Supplementary Figure S3. Comparable frequencies of M2 macrophages between PI MF- and PI MF+ cohorts. To analyze if MF can influence macrophage populations after start of treatment, the previously infected (PI) group was stratified according to presence or absence of MF after implementation and intensification of MDA in 2001 and 2009, respectively. Peripheral whole blood cells from endemic normal (EN; n = 89), latent (CFA+MF-; n = 54) and patent (CFA+MF+; n = 14) *Wuchereria bancrofti*-infected as well as previously infected individuals without (PI MF-; n = 86) or with MF (PI MF+; n = 31) before intensification of MDA programs and individuals that suffer from lymphedema (LE; n = 85) were analyzed for frequencies (%) of (A) CD14+ cells, (B) M2 macrophages (HLA-DR-CD206+CD163+CD200R+) and M2 macrophages expressing (C) IL-4 or (D) IL-10. Graphs show box whiskers with median, interquartile ranges and outliers (dots). Statistical significances between the indicated groups were obtained after a Kruskal-Wallis-test followed by a Dunn‘s multiple comparison post hoc analysis.

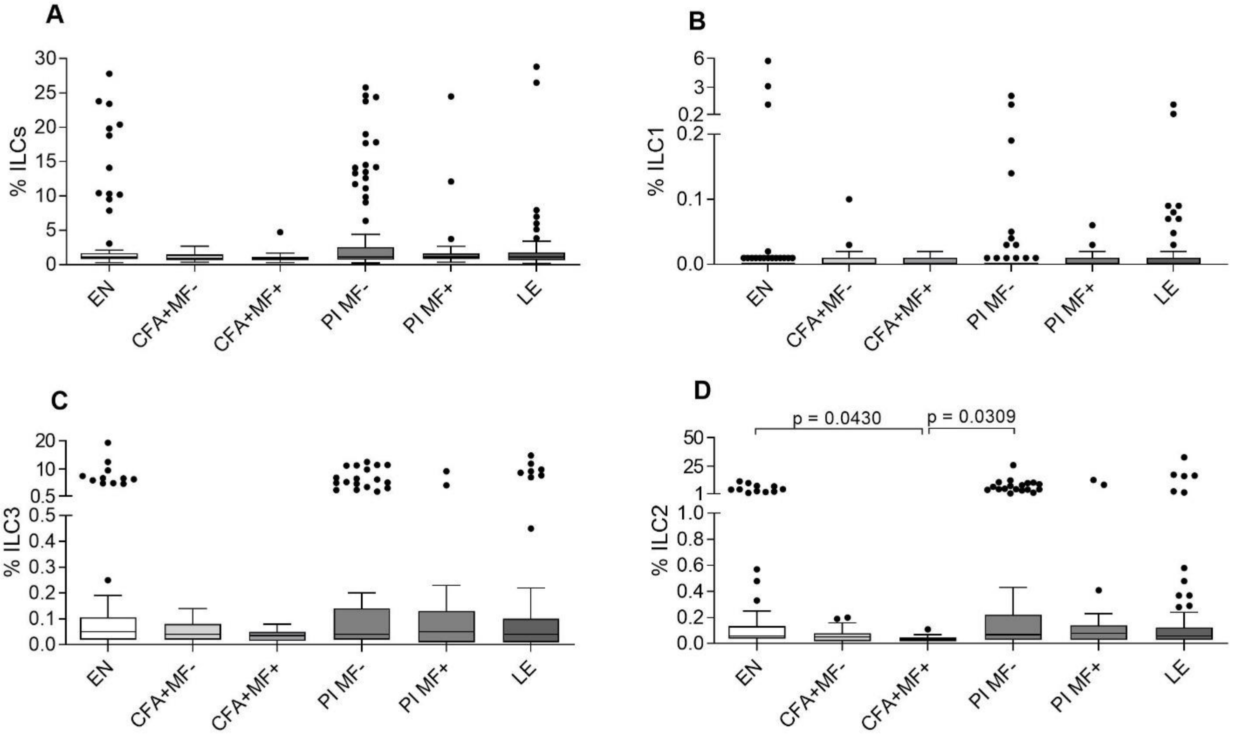

Supplementary Figure S4. Former MF status does not influence recovery of ILC2 frequencies in the PI cohort. To analyze if MF can influence ILC composition also after start of treatment, the previously infected (PI) group was stratified according to presence or absence of MF after implementation and intensification of MDA in 2001 and 2009, respectively. Peripheral whole blood cells from endemic normal (EN; *n* = 89), latent (CFA+MF-; *n* = 54) and patent (CFA+MF+; *n* = 14) *Wuchereria bancrofti*-infected as well as previously infected individuals without (PI MF-; *n* = 86) or with MF (PI MF+; *n* = 31) before intensification of MDA programs and individuals that suffer from lymphedema (LE; *n* = 85) were analyzed for frequencies (%) of (A) total ILCs (Lin-CD127+SSC-), (B) ILC1 (Lin-CD127+CD294-CD117-), (C) ILC3 (Lin-CD127+CD294-CD117+) and (D) ILC2 (Lin-CD127+CD294+). Graphs show box whiskers with median, interquartile ranges and outliers (dots). Statistical significances between the indicated groups were obtained after a Kruskal-Wallis-test followed by a Dunn's multiple comparison post hoc analysis.
